# A new model for predicting the winner in tennis based on the eigenvector centrality

**DOI:** 10.1007/s10479-022-04594-7

**Published:** 2022-03-07

**Authors:** Alberto Arcagni, Vincenzo Candila, Rosanna Grassi

**Affiliations:** 1 MEMOTEF Department, Sapienza University of Rome, Rome, Italy; 2 Department of Statistics and Quantitative Methods, University of Milano-Bicocca, Milan, Italy

**Keywords:** Network, Eigenvector centrality, Tennis, Forecasting

## Abstract

The use of statistical tools for predicting the winner in tennis matches has enjoyed an increase in popularity over the last two decades and, currently, a variety of methods are available. In particular, paired comparison approaches make use of latent ability estimates or rating calculations to determine the probability that a player will win a match. In this paper, we extend this latter class of models by using network indicators for the predictions. We propose a measure based on eigenvector centrality. Unlike what happens for the standard paired comparisons class (where the rates or latent abilities only change at time *t* for those players involved in the matches at time *t*), the use of a centrality measure allows the ratings of the whole set of players to vary every time there is a new match. The resulting ratings are then used as a covariate in a simple logit model. Evaluating the proposed approach with respect to some popular competing specifications, we find that the centrality-based approach largely and consistently outperforms all the alternative models considered in terms of the prediction accuracy. Finally, the proposed method also achieves positive betting results.

## Introduction

The advent of big data has significantly favoured the application of statistical tools in sports. From this aspect, Morgulev et al. (2018) presented a survey of data-driven analyses for a variety of sports, before and since the advent of the computerized era. The increasing popularity of statistical tools to forecast the outcomes of sport events is facilitated by at least two factors. First, the outcome is observable (contrary to what happens for predictions in financial markets, where the variable of interest may be latent, i.e. the volatility, and a proxy has to be used). Second, the vast number of variables potentially influencing the outcome of a match enriches the set of statistical methods at our disposal.

In the literature aimed at predicting the outcomes of sporting events, soccer (Koopman and Lit 2015; Angelini and De Angelis 2017; Mattera 2021, among others) and tennis play a prominent role. Regarding the latter, Kovalchik (2016) identifies three main categories of statistical methods used to forecast the winner of the match, namely regression-based (see, for instance, Del Corral and Prieto-Rodriguez 2010; Lisi and Zanella 2017), point-based (Barnett and Clarke 2005; Knottenbelt et al. 2012, among others), and paired comparisons (like the Bradley-Terry-type model of McHale and Morton 2011) approaches. Recently, the methods for predicting the probability of winning in tennis have been further extended. For example, artificial neural networks have been considered by Cornman et al. (2017) and Candila and Palazzo (2020). Within the paired comparison class of models, some weighted versions of the popular Elo method have been proposed, as in the contributions of Kovalchik (2020) and Angelini et al. (2022).

Although paired comparison methods seem to provide consistently better forecasts than other models (as reported, for instance, by Kovalchik 2016, 2020; Angelini et al. 2022), they have a not insignificant drawback–the latent ability, also defined rating, of players is calculated before each match. Thus, the probability of winning for the upcoming match is calculated using the previously obtained abilities. The rating of a player is only updated if that player participates in a match. In other words, when a player does not face any competitor, his/her rating remains constant. Hence, such models do not allow for dynamic updates to the ability/rating of a player, when the player is not playing. The aim of this paper is to fill this gap. In particular, for the first time, we compute the abilities/ratings in a network context. In this framework, every new match serves to update the full network, rather than only the ratings of the players involved. Network theory offers an effective tool for describing and capturing the ability of players (see Brandão et al. 2015; Arriaza-Ardiles et al. 2018). A directed network can accurately describe the outcomes of matches, where nodes denote players and a weighted link from one player to another quantifies the number of lost matches. In the context of complex networks, we focus on centrality measures, and specifically on the eigenvector centrality (Bonacich 1972). Indeed, the characteristic of this measure is that a node is highly central when it is connected to nodes which are themselves central, that is a node has a high score when is connected to nodes having themselves a high score. This measure is then particularly effective in representing the quality of players. This concept has been explored in the literature by Radicchi (2011), who used the PageRank to identify the best player from the records of professional sport competitions. By means of a particular network that incorporates a temporal memory of the matches, we propose a new measure based on the directed version of the eigenvector centrality, the so-called Bonacich centrality (Bonacich and Lloyd 2001).

Once the ratings have been calculated, we obtain the probability of winning for the upcoming match through a simple logit model. We evaluate the proposed model over two large datasets covering 14,170 male and 15,181 female matches over the period from 2016 to 2020. In terms of results, the eigenvector centrality-based model has a superior and statistically significant forecasting ability with respect to other models, independent of the year and type of dataset (i.e. male or female matches). The other models compared in this work include regression-based and paired comparison approaches. In particular, the competing models are the standard Elo model (Elo 1978); a recently proposed Elo-based model with the margin of victory (Kovalchik 2020); the logit-based models of Klaassen and Magnus (2003), Boulier and Stekler (1999), and Clarke and Dyte (2000); and, the Bradley-Terry-type model of McHale and Morton (2011). We also evaluate the proposed model from a betting perspective, by evaluating the gains or losses of the centrality-based specification with respect to the other models.

The remainder of this paper is structured as follows. In Sect. 2, we propose a new measure for determining the players’ rating. Section 3 reports the empirical analysis. We evaluate the players’ ratings and use previous results to predict the winning probabilities. The results achieved by our model are compared with those of the other models. Finally, the conclusions of this study are summarized in Sect. 4.

## A network approach for players ratings

Some definitions regarding directed graphs are essential before we introduce our measure for players ratings. The appendix gives these definitions in detail.

As the mathematical object representing the network is a graph, in the rest of this paper, we will refer to the network using the words “network” or “graph” interchangeably.

Let *i* and *j* be two generic players among the *n* different available players. Moreover, let $t_0,t_1,t_2,\ldots ,t_T$ denote the successive points in time at which the matches between the players are collected. Let *t* indicate the time at which a set of matches is disputed. Such notation is useful for characterizing the time series nature of the ratings, which will be described below.

The proposed rating system we propose is based on a centrality measure of a particular digraph $G=(V, A)$, where *V* is the set of *n* nodes, representing the unique players, and *A* is the set of arcs. Moreover, this digraph evolves over time, and its evolution determines the *n* time series (one for each player) of the rating system. We focus on the definition of the digraph at a given time *t*, $G_t=(V_t, A_t)$. We assume that the set of nodes *V* of the digraph is constant in time and corresponds to the players, and that arc (*i*, *j*) goes from *i* to *j* if the player *i* loses against the player *j*. In fact, the direction of the arc can be interpreted as a skill transfer from the losing player toward the winning player. Through this approach, a stronger player has more corresponding incoming arcs.

In relation to the centrality definition, a stronger player is one who wins more matches. Moreover, we apply the Bonacich centrality to graph $G_t$, which returns high scores for players that win against other players with high scores. Such scores are normalized so that they are comparable in time and in different digraphs (see the appendix for the precise definition).

Consider that, before the time *t*, player *i* may have lost more than once against *j*. Therefore, we introduce a function $c_t$ to digraph $G_t$, representing the weights of the arcs, $G_t = (V, A_t, c_t)$, where $c_t$ represents the number of lost matches in the time interval $[t_0, t]$, with $t_0$ denoting the origin of the observation time. This approach is in line with Radicchi (2011).

An important property of the Bonacich centrality of the digraph $G_t$ is that it depends on the whole network and if, for instance, a player stops playing for a period, then his/her score decreases. On the contrary, other rating systems remain constant. Moreover, in the application of the Bonacich centrality, young and emerging players are penalized due to the shorter observation time. Thus, we weigh the matches in inverse proportion to their oldness.

Therefore, the final definition of the function $c_t$ is provided through the corresponding weighted adjacency matrix:

1 $$\begin{aligned} {\mathbf {W}}_t = \sum _{t^* \in [t_0, t]} f(t^*, t, \alpha ) \cdot {\mathbf {L}}_{t^*}, \end{aligned}$$

where ${\mathbf {L}}_{t^*}$ is the $n \times n$ (corresponding to the *n* players) matrix of the number of lost matches exactly at time $t^* \in [t_0, t]$ and

2 $$\begin{aligned} f(t^*, t, \alpha ) = \left( 1 + \frac{t - t^*}{\alpha }\right) ^{-1} \text { with } t^* \in [t_0, t], \alpha > 0. \end{aligned}$$

The parameter $\alpha$ is a “memory” parameter; higher values of $\alpha$ assign higher weights to the oldest matches. In particular,

$$\begin{aligned} \lim _{\alpha \rightarrow +\infty } f(t^*, t, \alpha ) = 1\, \forall t \text { and } t^* \in [t_0, t] \end{aligned}$$

and all the matches have the same weight. Moreover, Eq. (2) decreases as the difference between *t* and $t^*$ increases. In particular, if the difference is 0 then $f(t, t, \alpha ) = 1$, and if it is $\alpha$ then $f(t - \alpha , t, \alpha ) = 0.5$. This last observation helps in choosing a suitable value of $\alpha$ that can be interpreted as the time distance in which the relevance of the matches is halved.

Note that if a player does not play, his/her score depends on the memory of function *f*, but it is also captured by the network context. In fact, if the player *j* stops playing, his/her weights $w_{ij}$ do not increase, differently from the other players. Under the proposed measure, however, the Bonacich centrality captures the weights variations of the whole network.

In conclusion, we summarize the steps involved in computing and interpreting the proposed measure. As it is based on the Bonacich centrality, we call this measure the *B-score*.

1. Construction of the matrix ${\mathbf {L}}_{t^*}$ of the number of lost matches
2. Construction of the matrix ${\mathbf {W}}_t$ associated with the digraph $G_t$, as defined in Eq. (1);
3. Calculation of the B-score at time *t* for each player by computing the Bonacich centrality of the matrix ${\mathbf {W}}'_t$ (see Eq. (11) in the appendix for details);
4. Repeat steps 1-3 for all *t*.

## Empirical analysis

The data used in this work were collected from the site www.tennis-data.co.uk, which provides a large number of statistics concerning the match results of professional tennis players. This archive has recently been used also by Angelini et al. (2022) and Baker and McHale (2017), among others. We use both the male and female archives. The information included are the match scores of all the most important tennis tournaments, the (closing) betting odds of different professional bookmakers, and the official rankings and the points for each player at the time of the match. The sample under investigation covers the period from July 4, 2005, to November 22, 2020, for the male’s matches and the period from January 1, 2007, to October 25, 2020, for the female’s matches. We have restricted the sample to the matches involved in the Masters 1000 (governed by the Association of Tennis Professionals (ATP)) and Premier (organized by the Women’s Tennis Association (WTA)) tournaments, as well as in the Grand Slams. After running the *clean* function of the R package ‘welo’ (Candila 2021), 14,170 male and 15,181 female matches remained. Overall, the data include 470 male and 455 female players.

At the beginning of the analysis, the full sample was divided into two non-overlapping periods: a training sample (from 2005 to 2015, consisting of 10,029 male and 9654 female matches) and a testing sample (from 2016 to 2020, that is, 4141 male and 5527 female matches). The training sample was used to estimate all the models predicting the probability of the winner, to which we dedicate the next subsection. The testing period was the out-of-sample period, which was used to evaluate the performances of the set of models. The process for obtaining the probability of winning is described by the following algorithm:

1. Estimate all the models from the beginning of the sample up to 2015.
2. Using the estimated parameters, obtain the probability of winning for the following 300 matches.
3. Add the matches of the previous step to the estimation sample. Re-estimate all the models and calculate the probability of winning for the following 300 matches.
4. Repeat steps 2 and 3 until the end of the sample.

### Application of the centrality-based measure

**Fig. 1:**
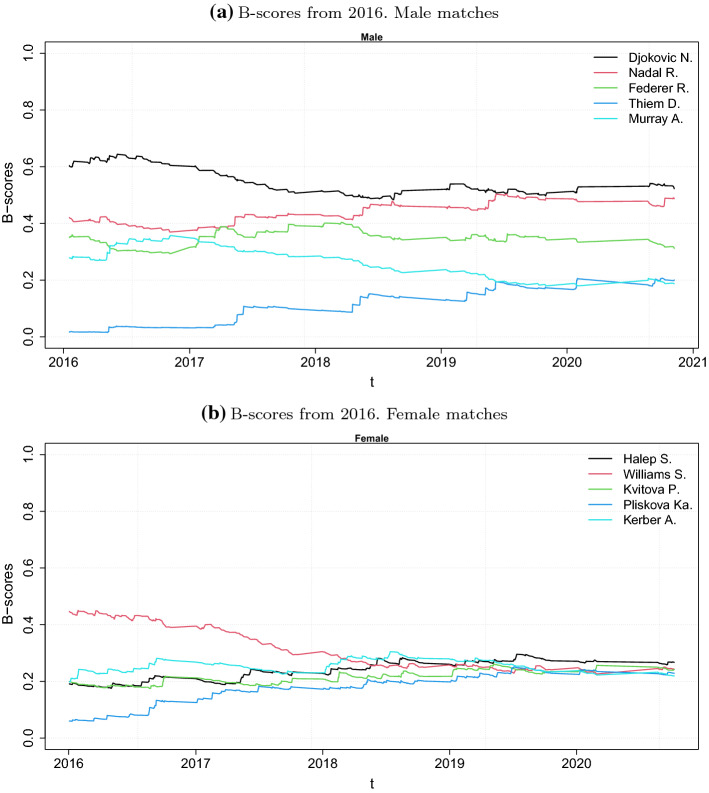
B-scores from 2016

Figure 1 shows the evaluation of the B-scores for the five players that have the highest scores at the end of the sample period for both the male and female circuits.

We only present the B-scores for the test sample period, as described at the beginning of this section.

As the Euclidean norm of the scores is one, they can be compared in time and between circuits. First, note that the maximum score that can be reached is one. Moreover, the second moment is constant, and so the variability is inversely proportional to the square of the average value. In the last years of the sample period, the average value in the male circuit is greater than that for the female circuit. Thus, it can be concluded that the female circuit is more variable, even if this cannot be observed from the plots because only the top five are shown.

Observe the decreasing trend of Andy Murray’s B-scores starting from 2017. The measure catches his hip injury and his retirement from the remainder of the season. This can be observed because the scores depend on the whole network at time *t* and because older matches are assigned lower weights in the network definition.

We used $\alpha =365$ days, in line with the ATP and WTA ranking systems, where the points earned during an ATP or WTA approved tournament remain in the system for 52 consecutive weeks.^1^

Finally, the normalization of the measure allows us to observe that the centrality of Serena Williams in the female circuit at the beginning of the testing sample period is similar to that of Rafael Nadal in the male circuit.

### Competing models for the winning probabilities

The competing models used in this work are: the standard Elo model (Elo 1978); the models, based on the logit regression, proposed by Klaassen and Magnus (2003), Boulier and Stekler (1999), and Clarke and Dyte (2000), labelled as (KM-reg), (BS-reg) and (CD-reg), respectively; and the Bradley-Terry-type model (BT-m) of McHale and Morton (2011). As mentioned above, we compute the probability of winning starting from the B-scores by means of a logit model. The set of models is briefly described in Table 1.

**Table 1:** Competing models

Label	Full name	Source
B-sc	B-score	
Elo	Elo	Elo (1978)
Lin-Elo	Linear Elo	Kovalchik (2020)
J-A-Elo	Joint Additive Elo	Kovalchik (2020)
Mult-Elo	Multiplicative Elo	Kovalchik (2020)
Log-Elo	Logistic Elo	Kovalchik (2020)
KM-reg	Logit regression	Klaassen and Magnus (2003)
BS-reg	Logit regression	Boulier and Stekler (1999)
CD-reg	Logit regression	Clarke and Dyte (2000)
BT-m	Bradley-Terry model	McHale and Morton (2011)

The table illustrates the set of competing models

In the following, we describe the process through which we transform the B-scores into probabilities. The other specifications employed in this work are then discussed.

The proposed centrality-based model returns the rates for players *i* and *j* for each match at time *t*, denoted by $CR_{i,t}$ and $CR_{j,t}$, respectively. $CR_{i,t}$ and $CR_{j,t}$ are observed after the end of the match between players *i* and *j*. These rates are then converted into probabilities through a simple logit regression, where $CR_{i,t}$ and $CR_{j,t}$ are the covariates used to predict the probability $p_{i,j,t+1}$, that is, the probability that player *i* wins over player *j* for a match at time $t+1$ (the probability that player *j* wins over player *i*, that is, $p_{j,i,t+1}$, is obtained as the complement to one of $p_{i,j,t+1}$). Formally,

3 $$\begin{aligned} p_{i,j,t+1} = \frac{\mathrm{exp}(\beta _0 + \beta _1 CR_{i,t} + \beta _2CR_{j,t})}{1+\mathrm{exp}(\beta _0 + \beta _1 CR_{i,t} + \beta _2CR_{j,t})}. \end{aligned}$$

Let $E_{i,t}$ and $E_{j,t}$ be the Elo ratings for the match at time *t* for players *i* and *j*, respectively. Then, the probability that player *i* defeats player *j* in a match at time *t* is:

4 $$\begin{aligned} {p}_{i,j,t} = \frac{1}{1+10^{\left( E_{j,t}-E_{i,t}\right) /400}}. \end{aligned}$$

The updating procedure for obtaining the Elo ratings for player *i* (and similarly for player *j*) is:

5 $$\begin{aligned} E_{i,t+1} = E_{i,t} + K_{i,t} \left[ W_{i,t}- {p}_{i,j,t} \right] , \end{aligned}$$

where $W_{i,t}$ is an indicator function equal to one if player *i* wins the match at time *t* and zero otherwise, and $K_{i,t}$ represents a scale factor. This scale factor expresses how much the Elo rating varies after the match at time *t*. According to Kovalchik (2016), the scale factor is assumed to depend on the number of matches. This means that the scale factor varies less when player *i* has played more matches. Recently, Kovalchik (2020) extended the Elo ratings by incorporating the margin of victory. She proposed four additional Elo rates: the Linear Elo (Lin-Elo), Joint Additive Elo (J-A-Elo), Multiplicative Elo (Mult-Elo), and Logistic-Elo (Log-Elo). For further details on these models, see Kovalchik (2020).

For the other models employing the logit regression, we use the following standard notations. Let $F_{i,t}$ and $F_{j,t}$ be, respectively, a feature of players *i* and *j* for the match at time *t*. All these logit-based models define

6 $$\begin{aligned} D_t = {F}_{i,t} - {F}_{j,t} \end{aligned}$$

as the regressor to include in the equation

7 $$\begin{aligned} p_{i,j,t} = \frac{\mathrm{exp}(\lambda D_t)}{1+\mathrm{exp}(\lambda D_t)}, \end{aligned}$$

where $p_{i,j,t}$ is the probability of winning for player *i* over player *j* at time *t* and $\lambda$ is the parameter to be estimated.

The KM-reg model defines the feature *F* as a logarithmic transformation of the rankings of the players ($R_{i,t}$ and $R_{j,t}$), that is:

$$\begin{aligned} {F}_{i,t}= 8 - 2 \log _2\left( R_{i,t}\right) , \end{aligned}$$

and

$$\begin{aligned} {F}_{j,t}= 8 - 2 \log _2\left( R_{j,t}\right) . \end{aligned}$$

The BS-reg model considers *F* to be the ranking at time *t*, without making any type of transformation. Hence, $D_t=R_{i,t}-R_{j,t}$.

The CD-reg model defines the feature *F* as:

$$\begin{aligned} {F}_{i,t}= \log \left( P_{i,t}\right) , \end{aligned}$$

and

$$\begin{aligned} {F}_{j,t}= \log \left( P_{i,t}\right) , \end{aligned}$$

where $P_{i,t}$ and $P_{j,t}$ are the points gained by players *i* and *j* at time *t*.

The BT-reg model calculates the probability of winning of player *i* beating player *j* as a function of (past) ability to win a game. Let $\alpha _{i,j,t}$ and $\alpha _{j,i,t}$ be the abilities of player *i* to win a game over *j* and vice versa, in the match at time *t*. The probability of player *i* winning the match at time *t*, denoted as $pg_{i,j,t}$, is:

8 $$\begin{aligned} pg_{i,j,t}= \frac{\alpha _{i,j,t}}{\alpha _{i,j,t}+\alpha _{j,i,t}}. \end{aligned}$$

The abilities for all the players are obtained by maximizing the likelihood provided in McHale and Morton (2011) (see their Eq. (1)). Once the abilities for each player have been estimated, it is then possible to calculate the probabilities of winning the match.

### Statistical evaluation

The loss functions used in this work to evaluate the forecasts in this work are the Brier Score (BS, Brier 1950) and the log-loss. Further forecast evaluation methods in sports are discussed in Reade et al. (2021). As in Angelini et al. (2022) and Gorgi et al. (2019), we use the Diebold and Mariano (1995) (DM) test to statistically assess the performance of the proposed model with respect to each competing specification. The results of this evaluation are presented in Tables 2 (male matches) and 3 (female matches). The tables illustrate the averages of the losses, for each model, and the DM test statistics with their significance. Notably, the DM test statistic is built on the difference between the losses of the proposed model and each competing specification. Therefore, if the test statistic is negative, it means that, on average, the losses produced by the B-scores are smaller than those of the competing model. Interestingly, the null hypothesis of equal predictive ability is almost always rejected, meaning that the proposed model largely outperforms all the other models. These results hold independently of the single year within the out-of-sample period considered, the loss used, and the male or female set of matches under investigation. Finally, the proposed model always achieves superior predictive ability for the full out-of-sample period.

**Table 2:** Centrality-based model evaluation against competing models according to DM test (male matches)

		# Matches	B-sc	Elo	Lin-Elo	J-A-Elo	Mult-Elo	Log-Elo	KM-reg	BS-reg	CD-reg	BT-m
Brier Score	2016	933	0.179	0.188	0.191	0.19	0.189	0.203	0.189	0.19	0.212	0.199
				[$$-2.113^{**}$$]	[$$-2.652^{***}$$]	[$$-2.477^{**}$$]	[$$-2.4^{**}$$]	[$$-4.207^{***}$$]	[$$-2.364^{**}$$]	[$$-2.494^{**}$$]	[$$-5.753^{***}$$]	[$$-3.863^{***}$$]
	2017	924	0.201	0.213	0.208	0.209	0.211	0.227	0.207	0.207	0.215	0.21
				[$$-2.702^{***}$$]	[$$-1.641$$]	[$$-1.915^{*}$$]	[$$-2.233^{**}$$]	[$$-4.46^{***}$$]	[$$-1.394$$]	[$$-1.307$$]	[$$-2.471^{**}$$]	[$$-1.748^{*}$$]
	2018	884	0.192	0.206	0.204	0.203	0.203	0.215	0.211	0.21	0.219	0.217
				[$$-3.162^{***}$$]	[$$-2.627^{***}$$]	[$$-2.538^{**}$$]	[$$-2.569^{**}$$]	[$$-4.104^{***}$$]	[$$-3.567^{***}$$]	[$$-3.365^{***}$$]	[$$-4.857^{***}$$]	[$$-4.268^{***}$$]
	2019	959	0.208	0.215	0.216	0.215	0.215	0.236	0.213	0.214	0.223	0.222
				[$$-1.53$$]	[$$-1.794^{*}$$]	[$$-1.567$$]	[$$-1.617$$]	[$$-4.676^{***}$$]	[$$-1.008$$]	[$$-1.221$$]	[$$-2.649^{***}$$]	[$$-2.468^{**}$$]
	2020	441	0.184	0.204	0.203	0.202	0.202	0.215	0.207	0.207	0.221	0.205
				[$$-2.854^{***}$$]	[$$-2.806^{***}$$]	[$$-2.712^{***}$$]	[$$-2.73^{***}$$]	[$$-3.407^{***}$$]	[$$-3.71^{***}$$]	[$$-3.518^{***}$$]	[$$-4.933^{***}$$]	[$$-2.813^{***}$$]
	Full	4,141	0.194	0.206	0.205	0.204	0.205	0.22	0.205	0.205	0.218	0.211
				[$$-5.435^{***}$$]	[$$-5.041^{***}$$]	[$$-4.901^{***}$$]	[$$-5.063^{***}$$]	[$$-9.378^{***}$$]	[$$-5.125^{***}$$]	[$$-5.084^{***}$$]	[$$-8.962^{***}$$]	[$$-6.777^{***}$$]
Log-loss	2016	933	0.541	0.561	0.57	0.566	0.568	0.652	0.56	0.563	0.624	0.603
				[$$-1.795^{*}$$]	[$$-2.383^{**}$$]	[$$-2.135^{**}$$]	[$$-2.312^{**}$$]	[$$-5.025^{***}$$]	[$$-1.875^{*}$$]	[$$-2.036^{**}$$]	[$$-5.111^{***}$$]	[$$-3.599^{***}$$]
	2017	924	0.591	0.623	0.61	0.612	0.621	0.715	0.605	0.604	0.631	0.61
				[$$-2.791^{***}$$]	[$$-1.588$$]	[$$-1.839^{*}$$]	[$$-2.568^{**}$$]	[$$-5.744^{***}$$]	[$$-1.281$$]	[$$-1.17$$]	[$$-2.395^{**}$$]	[$$-1.556$$]
	2018	884	0.571	0.604	0.596	0.593	0.596	0.663	0.609	0.607	0.635	0.655
				[$$-3.063^{***}$$]	[$$-2.19^{**}$$]	[$$-2.047^{**}$$]	[$$-2.329^{**}$$]	[$$-4.809^{***}$$]	[$$-2.967^{***}$$]	[$$-2.805^{***}$$]	[$$-4.364^{***}$$]	[$$-4.494^{***}$$]
	2019	959	0.602	0.62	0.625	0.62	0.622	0.72	0.611	0.616	0.644	0.638
				[$$-1.672^{*}$$]	[$$-1.979^{**}$$]	[$$-1.675^{*}$$]	[$$-1.824^{*}$$]	[$$-5.996^{***}$$]	[$$-0.889$$]	[$$-1.249$$]	[$$-2.906^{***}$$]	[$$-2.627^{***}$$]
	2020	441	0.546	0.593	0.592	0.588	0.589	0.658	0.596	0.599	0.644	0.597
				[$$-2.784^{***}$$]	[$$-2.706^{***}$$]	[$$-2.619^{***}$$]	[$$-2.678^{***}$$]	[$$-3.845^{***}$$]	[$$-3.49^{***}$$]	[$$-3.376^{***}$$]	[$$-4.706^{***}$$]	[$$-2.723^{***}$$]
	Full	4141	0.573	0.601	0.599	0.597	0.6	0.685	0.596	0.598	0.635	0.623
				[$$-5.309^{***}$$]	[$$-4.727^{***}$$]	[$$-4.484^{***}$$]	[$$-5.142^{***}$$]	[$$-11.452^{***}$$]	[$$-4.441^{***}$$]	[$$-4.511^{***}$$]	[$$-8.424^{***}$$]	[$$-6.876^{***}$$]

The table reports the averages of the Brier and Log-loss functions, for each of the model in column. Numbers in square brackets are the Diebold–Mariano test statistics. Negative values mean that the proposed centrality-based model outperforms the model in column. Models’ definitions are in Table 1. $$^{*}$$, $$^{**}$$ and $$^{***}$$ denote significance at the 10%, 5% and 1% levels, respectively

**Table 3:** Centrality-based model evaluation against competing models accordint to DM test (female matches)

		# Matches	B-sc	Elo	Lin-Elo	J-A-Elo	Mult-Elo	Log-Elo	KM-reg	BS-reg	CD-reg	BT-m
Brier Score	2016	1238	0.213	0.22	0.218	0.218	0.218	0.233	0.221	0.22	0.224	0.233
				[$$-1.893^{*}$$]	[$$-1.321$$]	[$$-1.308$$]	[$$-1.484$$]	[$$-4.718^{***}$$]	[$$-2.161^{**}$$]	[$$-2.033^{**}$$]	[$$-2.77^{***}$$]	[$$-4.337^{***}$$]
	2017	1268	0.217	0.222	0.223	0.222	0.222	0.24	0.231	0.231	0.235	0.234
				[$$-1.41$$]	[$$-1.691^{*}$$]	[$$-1.32$$]	[$$-1.401$$]	[$$-5.088^{***}$$]	[$$-3.832^{***}$$]	[$$-3.736^{***}$$]	[$$-4.071^{***}$$]	[$$-3.985^{***}$$]
	2018	1273	0.214	0.218	0.217	0.217	0.219	0.235	0.223	0.222	0.227	0.246
				[$$-1.078$$]	[$$-0.849$$]	[$$-0.813$$]	[$$-1.397$$]	[$$-4.538^{***}$$]	[$$-2.214^{**}$$]	[$$-2.065^{**}$$]	[$$-2.811^{***}$$]	[$$-6.736^{***}$$]
	2019	1249	0.215	0.223	0.223	0.223	0.224	0.245	0.222	0.221	0.226	0.238
				[$$-2.223^{**}$$]	[$$-2.262^{**}$$]	[$$-2.282^{**}$$]	[$$-2.678^{***}$$]	[$$-6.417^{***}$$]	[$$-2.034^{**}$$]	[$$-1.733^{*}$$]	[$$-2.794^{***}$$]	[$$-4.956^{***}$$]
	2020	499	0.209	0.216	0.215	0.213	0.213	0.232	0.216	0.217	0.225	0.234
				[$$-1.117$$]	[$$-0.888$$]	[$$-0.658$$]	[$$-0.739$$]	[$$-3.038^{***}$$]	[$$-1.34$$]	[$$-1.596$$]	[$$-2.385^{**}$$]	[$$-3.294^{***}$$]
	Full	5527	0.214	0.22	0.22	0.219	0.22	0.238	0.223	0.223	0.228	0.238
				[$$-3.486^{***}$$]	[$$-3.185^{***}$$]	[$$-2.928^{***}$$]	[$$-3.548^{***}$$]	[$$-10.827^{***}$$]	[$$-5.294^{***}$$]	[$$-5.053^{***}$$]	[$$-6.667^{***}$$]	[$$-10.62^{***}$$]
Log-loss	2016	1238	0.616	0.63	0.628	0.626	0.628	0.703	0.632	0.63	0.64	0.689
				[$$-1.804^{*}$$]	[$$-1.446$$]	[$$-1.327$$]	[$$-1.559$$]	[$$-5.804^{***}$$]	[$$-2.139^{**}$$]	[$$-1.968^{**}$$]	[$$-2.756^{***}$$]	[$$-4.982^{***}$$]
	2017	1268	0.625	0.635	0.641	0.636	0.636	0.714	0.653	0.655	0.673	0.672
				[$$-1.299$$]	[$$-1.799^{*}$$]	[$$-1.313$$]	[$$-1.373$$]	[$$-5.897^{***}$$]	[$$-3.627^{***}$$]	[$$-3.646^{***}$$]	[$$-4.288^{***}$$]	[$$-3.834^{***}$$]
	2018	1273	0.619	0.628	0.629	0.627	0.632	0.715	0.637	0.636	0.652	0.772
				[$$-1.096$$]	[$$-1.169$$]	[$$-0.995$$]	[$$-1.621$$]	[$$-5.884^{***}$$]	[$$-2.141^{**}$$]	[$$-1.956^{*}$$]	[$$-2.857^{***}$$]	[$$-5.382^{***}$$]
	2019	1249	0.621	0.639	0.639	0.638	0.642	0.732	0.636	0.633	0.647	0.713
				[$$-1.972^{**}$$]	[$$-1.986^{**}$$]	[$$-1.964^{**}$$]	[$$-2.398^{**}$$]	[$$-6.935^{***}$$]	[$$-1.958^{*}$$]	[$$-1.526$$]	[$$-2.744^{***}$$]	[$$-5.167^{***}$$]
	2020	499	0.608	0.62	0.615	0.611	0.613	0.682	0.621	0.624	0.649	0.718
				[$$-0.818$$]	[$$-0.48$$]	[$$-0.223$$]	[$$-0.336$$]	[$$-3.149^{***}$$]	[$$-1.119$$]	[$$-1.384$$]	[$$-2.451^{**}$$]	[$$-3.332^{***}$$]
	Full	5527	0.619	0.632	0.632	0.63	0.633	0.713	0.638	0.637	0.653	0.712
				[$$-3.186^{***}$$]	[$$-3.195^{***}$$]	[$$-2.738^{***}$$]	[$$-3.425^{***}$$]	[$$-12.65^{***}$$]	[$$-5.049^{***}$$]	[$$-4.773^{***}$$]	[$$-6.804^{***}$$]	[$$-9.995^{***}$$]

The table reports the Diebold–Mariano test statistic. Negative values mean that the proposed centrality model outperforms the model in column. Models’ definitions are in Table 1. $$^{*}$$, $$^{**}$$ and $$^{***}$$ denote significance at the 10%, 5% and 1% levels, respectively

### Economic evaluation: betting opportunities

In this section, we describe the benefits of applying the centrality-based model on the betting markets. We use a simple betting strategy, on the basis of that described in Angelini et al. (2022) and the references therein. Let $o_{i,j,h,t}$ and $o_{j,i,h,t}$ denote the odds provided by professional bookmaker *h*, with $h=1,\ldots ,H$, for players *i* and *j*, respectively, at time *t*. Moreover, let $q_{i,j,h,t}$ and $q_{j,i,h,t}$ be, respectively, the implied probabilities for *i* and *j*, obtained as the reciprocal of the odds provided by bookmaker *h*:

$$\begin{aligned} q_{i,j,h,t}= \left( o_{i,j,h,t}\right) ^{-1} \quad \text {and}\quad q_{j,i,h,t}=\left( o_{j,i,h,t}\right) ^{-1}. \end{aligned}$$

To take benefit from the betting markets, we use the best odds available on the market at the time of the match. Hence, the best odds among the *H* bookmakers for player *i* are defined as:

9 $$\begin{aligned} o_{i,j,t}^{B}= \max _{h=1,\ldots ,H} o_{i,j,h,t}, \end{aligned}$$

while those for player *j* is:

10 $$\begin{aligned} o_{j,i,t}^{B}= \max _{h=1,\ldots ,H} o_{j,1,h,t}. \end{aligned}$$

The adopted betting strategy for player *i*, according to the thresholds *r* and *q*, is defined as follows:

#### Definition 1

The amount of 1 is placed on the best odds $o_{i,j,t}^B$ in Eq. (9) for player *i* for all the matches where it holds that

$$\begin{aligned} \quad {p}_{i,j,t}> r \quad \text {and} \quad q_{i,j,Bet365,t}>q, \end{aligned}$$

where $q_{i,j,Bet365,t}$ is the implied probability offered by the professional bookmaker Bet365.

The same betting rule is applied to player *j*, using $p_{j,i,t}$, $q_{j,i,Bet365,t}$, and $o_{j,i,t}^{B}$. For a given match at time *t*, the probability that the player *i* wins over player *j* can be smaller, equal to, or greater than the threshold *r*. From the bettor’s point of view, it would be convenient to bet on those matches where the probabilities of a certain player winning are high. The higher the value of *r*, the higher will be the probability of winning using a given model. From the other side, when *r* increases, a larger number of matches are excluded. Thus, the threshold *q* is used instead to exclude heavy underdogs. In particular, if $q = 0$, all the matches are considered. As *q* increases, the matches whose implied probabilities are smaller than the chosen value for *q* are excluded.

**Fig. 2:**
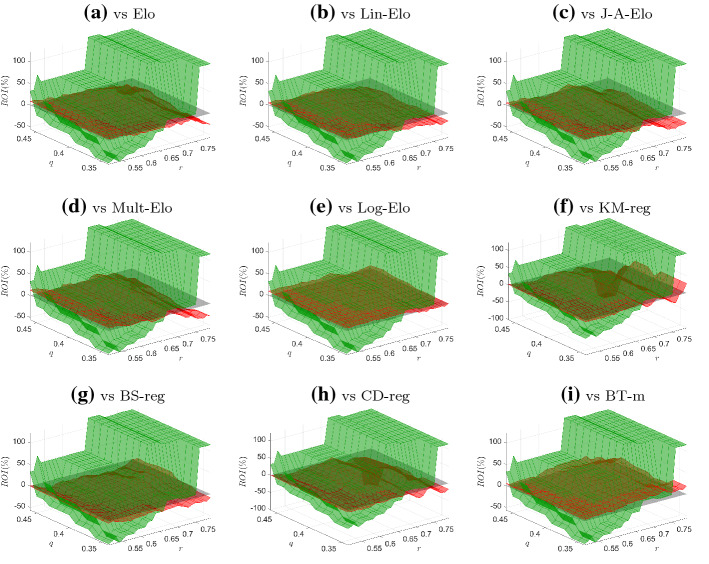
ROI for centrality-based and competing models (male matches). *Notes:* The figures depict the surfaces of the ROIs for the centrality-based model (green surface) against the ROIs of the competing models (red surface). Grey plane represents the case $\text {ROI}=0$. (Color figure online)

We applied this betting strategy to all the male and female matches in the test sample (from 2016 to 2020) and for all the probabilities obtained using the models synthesized in Table 1. The resulting return-on-investment (ROI), expressed in percentage terms, for different values of the thresholds *r* and *q* are reported in Figs. 2 (male matches) and 3 (female matches). These figures illustrate the surfaces (in green for the proposed model, in red for the competing specifications) as well as the grey plane denoting the case $\text {ROI}=0$. For the male matches, when $0.50< r < 0.60$, the centrality-based model gives a negative ROI, independently of the threshold *q*. However, when *r* is greater than 0.60, the ROI becomes to be positive and, in general, it is almost always larger than that of the competing models. For the female matches, the economic performance of the B-scores is largely better than the competing specifications: in fact, the green surfaces are almost always above the red surfaces, except for some combinations of very high *r* and *q* thresholds.

To conclude, the proposed centrality-based model appears to be superior to all the considered specifications, for the data and period under consideration, from a statistical and economic point of view.

**Fig. 3:**
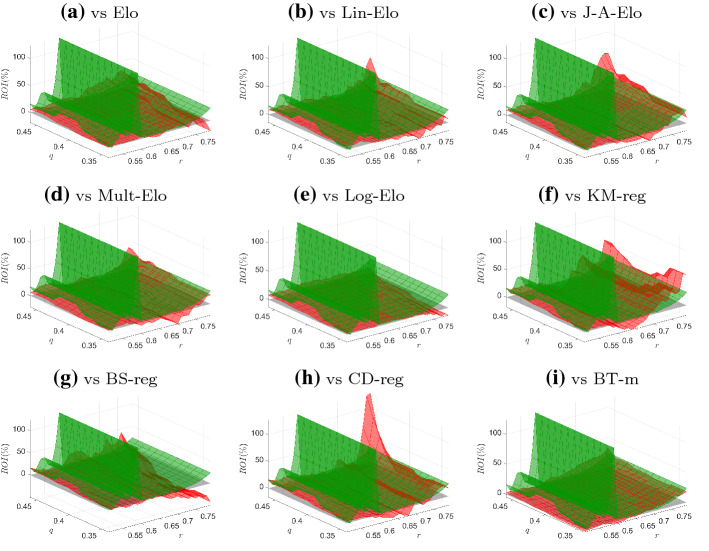
ROI for centrality-based and competing models (female matches).*Notes:* The figures depict the surfaces of the ROIs for the centrality-based model (green surface) against the ROIs of the competing models (red surface). Grey plane represents the case $\text {ROI}=0$. (Color figure online)

## Conclusions

In the big data era, statistical methods are increasingly used to predict the outcomes of sporting events. In this paper, we have described a method for computing the abilities/ratings of tennis players in a network context. By doing so, every new match updates the full network, rather than only the ratings of the players involved. We proposed a new measure called the B-score to rate the tennis players, who are considered as nodes of a network. The network incorporates the memory over time of previous matches, and the B-scores are obtained through the Bonacich centrality (Bonacich and Lloyd 2001). These scores are used in a logit regression model to determine the winning probabilities.

The B-scores take advantage of the network approach to better capture the evolution of each players’ ability. Specifically, for three tennis players *i*, *j* and *k* who have played each other at time $t-1$, if player *i* faces player *j* at time *t*, the standard paired comparison approach only updates the rates/abilities of players i and j. Player *k*’s rate remains unchanged, because *k* has not played any match at time *t*. In this situation, the proposed B-score allows the rate of *k* to vary at time *t* due to the dynamic relationship among all the players. In other words, the rate of *k* at time *t* changes because, in the past, player *k* has involved in a match against player *i* and/or player *j*.

The proposed approach has been extensively evaluated under two perspectives: fore casting ability and betting results. The forecasting ability was tested against several popular and recently developed alternatives.

Irrespectively of the period or tour (i.e. male of female matches) considered, the proposed approach has a statistically significant superiority in terms of forecasting ability with respect to all the competing models. In terms of betting returns, the proposed approach very often achieve superior ROIs with respect to all the other models under investigation.

In future research, the current centrality-based model will be expanded to account for different match conditions, such as the playing surface, through specific network structures. Also the choice of the parameter $\alpha$ is worthy of investigation, and further research may propose data-driven methods to calculate it. Finally, the current approach could be extended to other sports.

## Appendix

This appendix presents some definitions about graphs and networks, especially focusing on directed networks. This is intended to facilitate an understanding of the paper for readers who are not familiar with the mathematical framework.

### Graphs definitions

A network is a set of entities (agents) linked by pairwise relations. It is mathematically represented by a graph, the structure that models these relations. A graph is characterized by nodes connected by edges (or links). Graphs are undirected if the edges symmetrically connect two nodes; otherwise, the graph is said to be directed. We focus on directed graphs and provide some further definitions for them.

A directed graph or digraph is a graph in which the edges have an orientation. Formally, a directed graph (or digraph) $G=(V,A)$ is a pair of sets *V* and *A*, where *V* is the set of *n* nodes and *A* is the set of *m* ordered pairs (arcs) of nodes of *V*; if (*i*, *j*) and/or (*j*, *i*) is an element of *A*, then nodes *i* and *j* are adjacent. A directed walk of length $l \ge 1$ is any sequence of nodes $i_0,i_1,\ldots ,i_l$ such that $(i_{k-1},i_k) \in A, k=1,\ldots ,l$. Thus, an $i \rightarrow j$ directed walk is a sequence of nodes and arcs from *i* to *j* such that all arcs have the same direction. This definition leads to the idea of strong connectivity: a graph is strongly connected if any two nodes *i*, *j* are connected by (at least) an $i\rightarrow j$ directed walk.

A weight $w_{ij}>0$ can be associated with each arc (*i*, *j*) so that a weighted digraph is obtained. Formally, this is a directed graph *G* along with a mapping $c:A\rightarrow \Re$ such that $c(i,j)=w_{ij}$, that is a triple $G=(V,A,c)$. The elements $w_{ij}$ can be collected in a real *n*-square matrix ${\textbf {W}}$ (the *weighted adjacency matrix*) where the entries are $w_{ij}$ if $(i, j) \in A$ and zero otherwise. Note that, by definition, the elements of such a matrix describe both the adjacency relationships between the nodes in *V* and the weights on the arcs and, in general, $w_{ij} \ne w_{ji}$. In the unweighted case, nonnull weights can take only unitary values, so that the matrix ${\textbf {W}}$ provides only information about the adjacency relationships. In the case of unweighted networks, we denote ${\textbf {W}}$ by ${\textbf {A}}$ (the *adjacency matrix*).

An important result states that the graph *G* is strongly connected if and only if ${\mathbf {W}}$ is irreducible, and this allows the application of the *Perron-Frobenius Theorem*. Let $\{\lambda _1,\lambda _2,\ldots ,\lambda _n\}$ be the set of the eigenvalues of ${\mathbf {W}}$ and $\rho =max_i|\lambda _i|$ be its spectral radius. By the Perron-Frobenius Theorem, if ${\mathbf {W}}$ is irreducible and nonnegative, $\rho$ is an eigenvalue of ${\mathbf {W}}$ and there exists a positive eigenvector ${\mathbf {x}}$ associated with $\rho$. The vector ${\mathbf {x}}$ is called the *principal eigenvector*. See Horn and Johnson (2012) for a more detailed description of this concepts.^2^

### Centrality in networks

One of the major topics in network analysis concerns *centrality*. This concept, when applied to nodes, measures the importance of their position in the network.

Centrality does not have a unique definition, but depends on the characteristics of the network and the context. For instance, a node can be important because it has many connections, or conveys crucial information along paths, or is connected to nodes that are powerful themselves.

The first case (i.e. many connections) is the most intuitive and it is formally described by the degree centrality of a node *i*, which counts the number of edges incident to *i*. In a directed network this centrality measure is replaced by the out-degree and in-degree of a node *i*. In particular, the in-degree $d^{in}_i$ is the number of arcs arriving to the node *i* and the out-degree $d^{out}_i$ is the number of arcs starting from *i*.

Moving to weighted networks, the degree centrality is generalized by the strength centrality of the node *i*, which sums the weights of the edges incident to *i*. In the directed case, we define the in-strength centrality $s^{in}_i$ (out-strength centrality $s^{out}_i$) of *i* as the sum of the weights of all the edges coming to (going from) *i*.

The centrality measure that best represents the case in which a node *i* is central if connected to nodes that are central themselves is the eigenvector centrality (Bonacich 1972). For a node, this measure accounts for its connections, similar to the degree centrality. Indeed, the centrality scores of the node’s neighbours contribute to its centrality.

Mathematically, the measure is defined as the *i*-th component of the principal eigenvector of the matrix ${\textbf {A}}$, normalized with the Euclidean norm $\parallel {\mathbf {x}}\parallel _2$. Indeed, by the definition of the adjacency matrix ${\mathbf {A}}$, provided in the previous subsection, the score $x_i$ of the node *i* is proportional to the scores of its neighbours. The vector collecting the centrality scores is ${\textbf {x}}=\frac{1}{\rho }{} {\textbf {A}}{} {\textbf {x}}$.

The generalization of the eigenvector centrality to weighted digraphs can be introduced as follows. We assume that the entry $w_{ji} \ge 0$ means that the node *j* contributes (with weight $w_{ji}$) to *i*’s status, so the score of node *i* is proportional to the sum of scores of all nodes *j* pointing to *i* (see Bonacich and Lloyd 2001). Formally

11 $$\begin{aligned} {\textbf {x}}=\frac{1}{\rho }{} {\textbf {W}}'{} {\textbf {x}} \end{aligned}$$

Notice that, as the matrix ${\mathbf {W}}$ is not symmetric, the vector ${\mathbf {x}}$ (normalized with the Euclidean norm) is the principal eigenvector associated with $\rho$ of the irreducible matrix $\mathbf {W'}$. We refer to this centrality measure as Bonacich centrality.

In concluding this appendix, it is worth comparing the described measures in the weighted case, i.e. in-strength and Bonacich centrality. As done for the eigenvector centrality, we can collect the in-strength centrality into a vector:

$$\begin{aligned} {\mathbf {s}}^{in}=\mathbf {W'1} \end{aligned}$$

As already stressed, the *i*-th element of this vector is the sum of the weights of the nodes pointing to *i*. This means that its centrality $s^{in}_i$ only depends on the weights of its incoming links. On the contrary, by the Eq. (11), the centrality score of node *i* is proportional to the sum of the centrality scores of the nodes pointing to *i*, weighted with the entries of the matrix ${\mathbf {W}}$. In this sense, a node is highly central if pointed to by nodes that are highly central themselves. Thus, a powerful and strong node is determined by how strong and powerful are the nodes that point to it.

## Footnotes

1 Except during the first Covid-19 period, where the rule of 12-month ranking changed.
